# The Adsorption of Pb, Zn, Cu, Ni, and Cd by Modified Ligand in a Single Component Aqueous Solution: Equilibrium, Kinetic, Thermodynamic, and Desorption Studies

**DOI:** 10.1155/2017/6150209

**Published:** 2017-05-18

**Authors:** E. Igberase, P. Osifo, A. Ofomaja

**Affiliations:** Department of Chemical Engineering, Vaal University of Technology, Private Mail Bag X021, Vanderbijlpark 1900, South Africa

## Abstract

In this investigation, an amino functionalized adsorbent was developed by grafting 4-aminobenzoic acid onto the backbone of cross-linked chitosan beads. The 3 sets of beads including chitosan (CX), glutaraldehyde cross-linked chitosan (CCX), and 4-aminobenzoic acid grafted cross-linked chitosan (FGCX) were characterized by FTIR, XRD, SEM, and TGA. The water content and amine concentration of FGCX were determined. The effect of adsorption parameters was studied and the optimum was used for further studies. Equilibrium data was obtained from the adsorption experiment carried out at different initial concentration; the data were applied in isotherm, thermodynamics, and kinetic studies. The Langmuir and Dubinin-Kaganer-Radushkevich (DKR) models were successful in describing the isotherm data for the considered metal ions while the Freundlich and Temkin model fit some of the considered metal ions. Pseudo-second-order and intraparticle model described the kinetic data quite well. Thermodynamic parameters such as Gibb's free energy change (Δ*G*^*o*^), enthalpy change (Δ*H*^*o*^), and entropy change (Δ*S*^*o*^) were calculated and the results showed that the adsorption of Pb, Cu, Ni, Zn, and Cd ions onto FGCX is spontaneous and endothermic in nature. Regeneration of the spent adsorbent was efficient for the considered metal ions.

## 1. Introduction

Metals used across chemical, electroplating, leather, tannery, galvanizing, mining, pigment, and dye industries are regarded as the major toxic unbiodegradable elements present in the world today. This becomes a treat as the industrial effluents containing a reasonable amount of harmful metal ions are discharged into the environment without possible remediation which resulted in environmental issues; hence the environmental protection agency (USEPA) has considered certain metals as carcinogenic and bioaccumulative element. Among the most toxic metals are Cu, Cd, Pb, Zn, and Ni. These metals have been reported by researchers to have negative health effect on humans, aquatic life, plant, and the environment at large [1–4]. Various techniques have been applied for the removal of metal ions from industrial wastewaters including chemical precipitation, membrane separation, reverse osmosis, ion exchange, and nanofiltration [5]. These methods can be applied alone or integrating more than one method together, such as chemical precipitation prior to nanofiltration [1]. This procedure has been reported to be disadvantaged due to incomplete removal, high energy requirements, and production of toxic waste sludge which needs to be treated and disposed [6]. Recently, researchers have developed a more cost effective method to reduce or completely remove the metal ions to its allowable limit as given by USEPA. Thus, increasing the quality of the treated wastewater. Adsorption has proven to be of high standard due to its simple design and operation, low cost, effectiveness, and outstanding chelating behaviour and can be regenerated by some desorption techniques [7–9].

However, in providing cheaper and cleaner technology for the treatment of wastewater with toxic metal ions, chitosan is considered the solution. Chitosan is a derivative of N-deacetylation of chitin and chitin is a naturally occurring polysaccharide found in crustacean and microbial biomass [8]. The presence of amine (–NH_2_) and hydroxyl functional group in the back bone of chitosan gives the polymer its distinctive adsorption quality. Amine groups are mainly responsible for the binding of Cu, Cd, Pb, Zn, and Ni cations as shown in

(1) $$\begin{array}{c} \text{–}{NH_{3}}^{+}+M^{2+}⟶NH_{3}M^{2+}+H^{+} \end{array}$$

**Figure 1:**
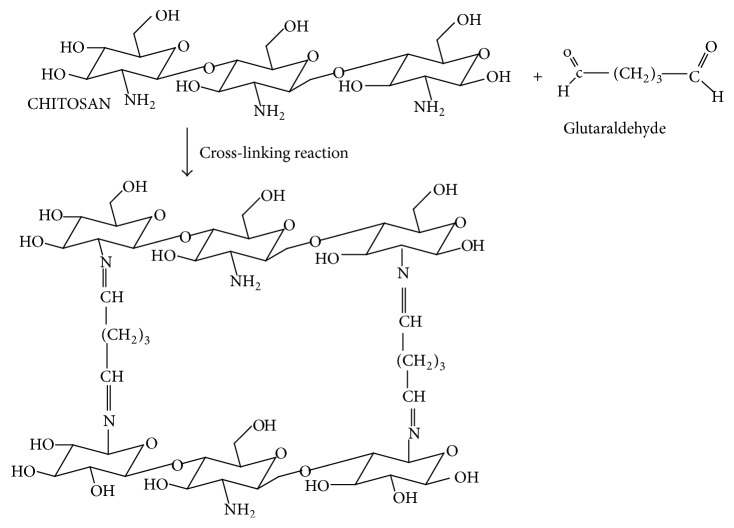
Schematic representation of the cross-linking process of chitosan.

**Figure 2:**
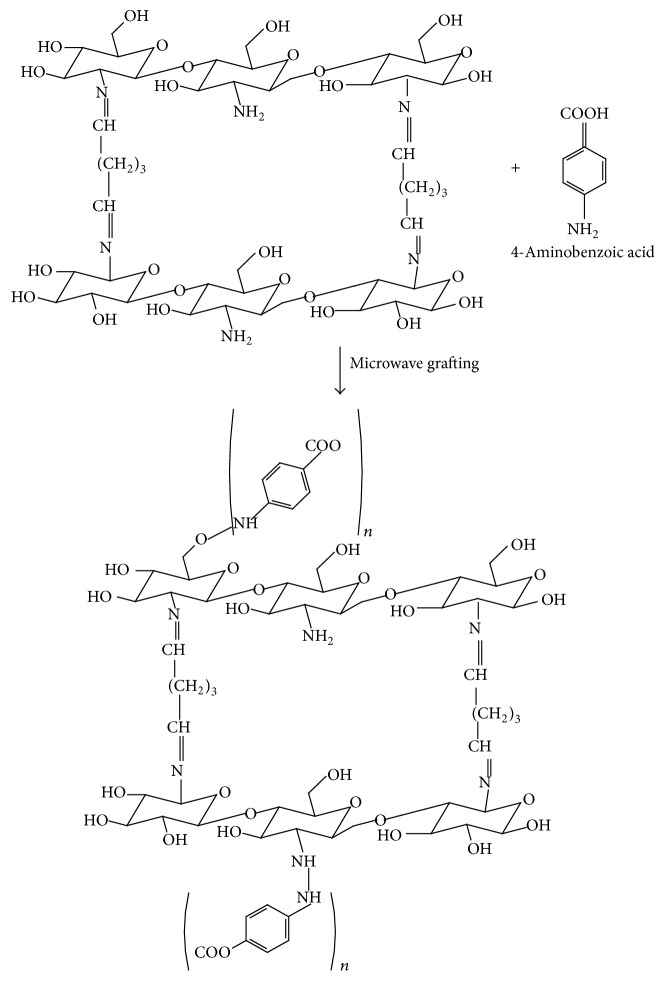
Schematic representation of the proposed structure of the grafting process of the cross-linked chitosan beads.

However, the adaptability of chitosan allows the polymer to be simply modified; hence, chemical modification techniques such as cross-linking and grafting become necessary. Cross-linking happens when a chemical or compound referred to as the cross-linker makes intermolecular covalent bridge between chains (Figure 1). The mechanism involves the formation of Schiff base via nucleophilic attack by the nitrogen of the amino group (from chitosan) on the carbon of the glutaraldehyde which displaces the oxygen of the aldehyde resulting in the C=N bond [10]. Cross-linking results in insolubility as the chains are attached together by strong covalent bond [11]. Consequently, cross-linking also has its short comings. Authors such as Gyananath and Balhal [12] and Igberase et al. [5] reported that cross-linking reduces the adsorption capacity of adsorbents Table 2; hence grafting of some functionality onto the cross-linked material becomes important. Grafting of chitosan allows the formation of functional derivatives by covalent binding of a molecule onto chitosan backbone.  Figure 2 shows the proposed grafting reaction of 4-aminobenzoic acid onto cross-linked chitosan. However, in this Figure the grafting material binds to the amino and hydroxyl group in the polymeric chain leading to a large number of adsorption sites.

The focus of this study is to consider the possible application of a modified ligand for the effective removal of metal ions in batch mode. On this note, chitosan solution was cross-linked with 2.5% glutaraldehyde concentration (Figure 1). The cross-linked solution was used in preparing the beads and then grafted with 4-aminobenzoic acid to produce FGCX (Figure 2). The water content amine concentration in the developed ligand was determined. The beads were characterized by Fourier transform infrared spectroscopy (FTIR), X-ray diffraction (XRD), thermogravimetric analysis (TGA), and scanning electron microscope (SEM) to provide evidence of successful modification process. The effect of adsorption parameters on the binding of metal ions onto FGCX was investigated. FGCX was used in adsorption studies. Adsorption/desorption studies were investigated in others to determine the cost effectiveness of the produced FGCX.

## 2. Experimental

### 2.1. Materials and Equipment

Materials used in this study were of analytical grade. Chitosan powder with a degree of deacetylation of 74% was obtained from China. The powder was used in preparing chitosan beads. Glutaraldehyde and 4-amino-benzoic acid (>99.5%) were purchased from Sigma-Aldrich. Domestic oven was used in the grafting of the cross-linked beads. Hydrochloric acid (>99%), acetic acid (>99%), and sodium hydroxide (>99%) were all purchased from Sigma-Aldrich. The pH of the solution was adjusted with a pH meter (Hanna HI 8421) and was purchased from Sigma-Aldrich. Distilled water was produced with a pure water distiller (Ultima 888 water distiller) in the school laboratory. A shaker (labcon incubator) was used for adsorption studies. Atomic adsorption spectrophotometer (Varian SpectrAA-10) was applied in determining the amount of metal ions adsorbed.

### 2.2. Adsorbate Preparation

The stock solutions of Cu^2+^, Cd^2+^, Zn^2+^, Pb^2+^, and Ni^2+^ were obtained by separately preparing a calculated mass of CuSO_4_·5H_2_O, CdCl_2_·H_2_O, Zn(NO_3_)_2_·6H_2_O, Pb(NO_3_)_2_, and NiSO_4_·6H_2_O. This stock solution produced was again diluted with distilled water to obtain the relevant initial concentrations of 0.5 to 2.5 mmol/l.

### 2.3. Preparation of Ligand and Possible Modification

Chitosan solution was prepared by dissolving 30 g of chitosan powder in 1 L of 5.0% (v/v) acetic acid solution. The dissolved solution was mixed with 2.5% glutaraldehyde solution and stirred with a magnetic stirrer for 2 hrs in others to achieve cross-linking reaction. The cross-linked solution was then passed through a glass pipette with the aid of a peristaltic pump to a 1 M solution of sodium hydroxide; this procedure results in the formation of glutaraldehyde-cross-linked chitosan gel beads. The gel beads were rinsed with distilled water several times to remove any residue sodium hydroxide. The beads produced by this method have mean diameter of 3.3 mm. Consequently, the cross-linked beads were grafted by microwave irradiation. This was actualised by mixing 4 g of the cross-linked beads in 0.1 g/l of 4-aminobenzoic acid in an open neck flask. This flask was then placed in a domestic microwave oven with a medium-low power for 20 mins. The grafted-cross-linked beads were again rinsed with distilled water and ready to be applied for studies. The water content (*W*_*C*_) present in the FGCX was determine using

(2) $$\begin{array}{c} W_{C}=\frac{W_{1}-W_{2}}{W_{2}}, \end{array}$$

where *W*_1_ and *W*_2_ denote the weights of wet FGCX and dry FGCX, respectively.

### 2.4. Determination of Amine Concentration of the Grafted-Cross-Linked Beads

10.0 g of the grafted cross-linked beads was weighed and ground with an 18 mm Teflon pestle; the grounded beads were mixed with distilled water up to a 100 ml. The solution was stirred continuously with a magnetic stirrer and titrated with 1 M of HCl. The pH of the solution was recorded at time interval; the result obtained from the experiment was used to determine the amine concentration in mmol/g of adsorbent.

### 2.5. Characterization of the Beads

Approximately 1.0 g of CX, CCX, and FGCX was separately weighed and oven dried at 60°C and blended to its powdered form. The infrared measurement was done with a Shimadzu FTIR model 8300 Kyoto, Japan; the spectra were recorded in the range of 500–4000 cm^−1^. The crystallinity of the beads was studied with a Shimadzu XRD model 7000; the intensities were recorded in the range of 10–90° (2*θ*). The weight loss of the beads at different temperatures was studied with a Shimadzu TGA 8000 Japan.

The SEM analysis was done by separately bisecting CX, CCX, and FGCX in other to get a distinct vision of the inner fibres. The bisected beads were then coated with gold and the morphology of the coated beads was studied with a Jeol 733 super probe.

### 2.6. Adsorption Studies

A known mass of FGCX was transferred into a series of Erlenmeyer flasks; then samples of 100 mL having a known concentration were measured into each flask. The pH of the samples was adjusted by 0.1 M HCl and 0.1 M NaOH. The effect of adsorption parameters was observed under the following conditions: pH (2–8), contact time (10–80) min, initial concentration (0.5–2.5) mmol/L, adsorbent dosage (2–10) g/L, temperature (25–55)°C, and ionic strength (0.05–0.2) M. The experiment was carried out in duplicate and the average result was shown in this study.

Isotherm studies were carried out by transferring 6 g/L of FGCX, separately into a series of 250 mL Erlenmeyer flasks; each of the flasks was filled with 100 mL of each metal solution with different initial concentrations of 0.5–2.5 mmol/L and at temperature of 45°C. These initial concentrations were prepared from a stock solution and then adjusted to optimum pH. In other to attain equilibrium, the Erlenmeyer flasks were placed in a labcon incubator for 60 min; the agitation speed was at 150 rpm. Thermodynamics was carried out at an initial concentration of 0.5 mmol/L. Kinetics was investigated upon by mixing 6 g/L of FGCX with 100 mL of metal ions solution in a series of 250 mL Erlenmeyer flasks having initial concentration of 0.5 mmol/L. In order to attain equilibrium, the Erlenmeyer flasks were placed in a labcon incubator, the solution was shaken at 150 rpm, and the temperature was fixed at 45°C. Samples were taken at interval of 10–60 min and analysed for metal ions removal. This procedure was observed for Pb, Cu, Ni, Cd, and Zn.

To determine the amount of metal ion adsorbed, the equilibrium adsorption capacity was calculated from the mass balance equation as shown in

(3) $$\begin{array}{c} q_{e}=\frac{\left(C_{o}-C_{e}\right)XV}{M}, \end{array}$$

where *q*_*e*_ (mmol/g) is the equilibrium adsorption capacity, *C*_*o*_ and *C*_*e*_ are the initial and equilibrium concentration (mmol/l) of heavy metal ion in solution, respectively, while *V* (mL) is the volume and *M* (g) is the weight of the adsorbent. The percentage removal of metal ions from the single component mixture was calculated by

(4) $$\begin{array}{c} \%\;\;R=\frac{\left(C_{o}-C_{e}\right)}{C_{o}}\times 100. \end{array}$$

### 2.7. Theory of Evaluation of Data

#### 2.7.1. Isotherm Model

Equilibrium is reached when the capacity of the binding material is achieved and the rate of binding corresponds with the rate of desorption [12]. Basically, the binding capacity of an adsorbent can be obtained from isotherms such as Langmuir, Freundlich, Temkin, and DKR model. The constants from the model are measures of binding capacity of adsorbent for the considered metal ions.

The Langmuir model is based on the fact that every adsorption site is identical and energetically equivalent and assumes that the adsorption occurs at specific homogeneous sites on the adsorbent and this is used successfully in monolayer adsorption processes [13]. This model is described in the linear form in

(5) $$\begin{array}{c} \frac{C_{e}}{q_{e}}=\frac{C_{e}}{Q_{m}}+\frac{1}{Q_{m}K_{L}}\;\left(\text{Linear form}\right). \end{array}$$

The Langmuir constant *Q*_*m*_ (mmol/g) represents the maximum adsorption capacity and *K*_*L*_ (l/mmol) relates to the rate of adsorption. Higher values of *K*_*L*_ indicate much stronger affinity of metal ion adsorption [14]. The parameters of the Langmuir model can be estimated from the slope and intercepts of *C*_*e*_/*q*_*e*_ versus *C*_*e*_. The basic characteristics of Langmuir model can be shown in terms of a dimensionless constant known as separation factor (*R*_*L*_) which is used to project if an adsorption system is favourable or not favourable [15], as shown in (6). The conditions of *R*_*L*_ > 1, *R*_*L*_ = 1 and *R*_*L*_ between 0 and 1 signify unfavourable, linear, and favourable, respectively,

(6) $$\begin{array}{c} R_{L}=\frac{1}{1+K_{L}C_{O}}. \end{array}$$

The empirical Freundlich isotherm is based on the equilibrium relationship between heterogeneous surfaces. This isotherm is derived from the assumption that the adsorption sites are distributed exponentially with respect to the heat of adsorption [16]. This model is described in the linear form in

(7) $$\begin{array}{c} \log q_{e}=\log K_{F}+\frac{1}{n}\log C_{e}\;\left(\text{Linear form}\right). \end{array}$$

*K*_*F*_ and *n* are constants representing the adsorption capacity and adsorption intensity, respectively. The parameters of this model can be calculated from the slope and intercepts of log⁡*q*_*e*_ versus log⁡*C*_*e*_ plot; under normal adsorption conditions, the values of *n* should be in the range of 1 to 10 [17].

The derivation of the Temkin model is based on the heat of adsorption of metal ions which assumes the relationship between metal ions and adsorbent is linear and is described by

(8) qe=RTbln⁡A+ln⁡Ce,

(9) RTb=B,

where *R* (8.314 Jmol/K) is the universal gas constant, *T* (K) is the temperature, and *C*_*e*_ (mmol/L) is the equilibrium concentration of metal ions. *A* (L/mmol) and *B* (J/mol) are Temkin isotherm constants associated with equilibrium adsorption constant and intensity of adsorption (9), respectively. A linear plot of amount adsorbed *q*_*e*_ versus ln⁡*C*_*e*_ gives the values of the constant *A* and *b* from the slope and intercept of the graph, respectively.

The unique feature of DKR model is that it is temperature dependent [18]; this model has been applied to study the adsorption of metal ions onto adsorbent [19]. The model equation is shown in

(10) $$\begin{array}{c} \ln q_{e}=\ln X_{m}-K_{ad}\varepsilon^{2}, \end{array}$$

where *K*_ad_ (mol^2^/KJ^2^) and *X*_*m*_ (mmol/g) are the DKR isotherm constants and *ε* is the Polanyi potential which is defined by

(11) $$\begin{array}{c} \varepsilon =RT\ln\left(\;1+\frac{1}{C_{e}}\;\right). \end{array}$$

The slope and intercept of the plot of ln⁡*q*_*e*_ versus *ε* give *K*_ad_ and *X*_*m*_, respectively. However, the adsorption energy required to remove each molecule of metal ions from its position into the adsorption site can be calculated using the relation in

(12) $$\begin{array}{c} E=\frac{1}{\sqrt{-2K_{ad}}}. \end{array}$$

The value of *E* is vital and can be applied in obtaining the nature of adsorption process; if the value of *E* is <8 kJ/mol, then the adsorption process is physical in nature but if it is between 8 and 16 kJ/mol then it can explain by ion exchange mechanism [20].

#### 2.7.2. Thermodynamic Parameters of Adsorption

The original ideal of thermodynamics is based on the assumption that in a system that is kept isolated, where energy cannot be gained or lost to the surroundings, the entropy change is the driving force. In environmental engineering practice, both energy and entropy factors must be considered to decide what processes will occur spontaneously [21]. The entropy and enthalpy change, associated with the process, can be calculated from

(13) $$\begin{array}{c} \ln K=-\frac{\Delta H^{o}}{RT}+\frac{\Delta S^{o}}{R}. \end{array}$$

The Gibbs free energy change, Δ*G*^*o*^, is the fundamental criterion of spontaneity. Reactions occur spontaneously at a given temperature if Δ*G*^*o*^ is a negative quantity [21]. The free energy of the adsorption reaction, considering the adsorption equilibrium constant, *K*, is given by

(14) $$\begin{array}{c} \Delta G^{o}=-RT\ln K. \end{array}$$

The equilibrium constant “*K*” as defined mathematically by Liu et al. [17] is given in

(15) $$\begin{array}{c} K=\frac{q_{e}}{C_{e}}. \end{array}$$

Δ*S*^*o*^ is the entropy change while Δ*H*^*o*^ is the enthalpy change. Δ*H*^*o*^ and Δ*S*^*o*^ can be calculated from the slope and intercept of a plot of ln⁡*K* as a function of 1/*T*.

### 2.8. Adsorption Kinetics

The evaluation of kinetics of a system can disclose the mechanism of binding. Most researchers apply the pseudo-first-order kinetics of Lagergren [22], the pseudo-second-order kinetic model that was introduced by Ho & McKay [23], and the intraparticle diffusion model as shown in (16)–(18) to describe kinetic data. These models are used to investigate the controlling mechanism of adsorption process

(16) $$\begin{array}{c} \log\left(\;q_{e}-q_{t}\;\right)=\log\left(\;q_{e}\;\right)-t\frac{K_{1}}{2.303}, \end{array}$$

where *q*_*e*_ and *q*_*t*_ represents the amount of copper ions absorbed on the adsorbent (mmol/g) at equilibrium and time *t*, respectively. *K*_1_ (min^−1^) is the rate constant of the pseudo-first-order kinetics. The value of adsorption rate constant, *K*_1_ can be calculated from the straight-line plot of log⁡(*q*_*e*_ − *q*_*t*_) versus *t*

(17) $$\begin{array}{c} \frac{t}{q_{t}}=\frac{1}{K_{2}q_{e}^{2}}+\frac{1}{q_{e}}t, \end{array}$$

where *K*_2_ (g/mmol·min) is the rate constant for a pseudo-second-order model and the definitions of *q*_*e*_ and *q*_*t*_ remain the same. The slope and intercept of the linear plot of *t*/*q*_*t*_ versus *t* give the values of *q*_*e*_ and *K*_2_, respectively.

Intraparticle diffusion model is very vital in that it is the rate determining step in any liquid adsorption system [14]. In a properly stirred batch adsorption system, the intraparticle diffusion model has been applied in analysing the adsorption process taking place in the porous adsorbent [24]. Intraparticle diffusion model varies directly with the rate constant and also the square root of time

(18) $$\begin{array}{c} q_{t}=K_{idm}\sqrt{t}, \end{array}$$

where *t* is the time (min) and *K*_idm_ (mmol/gmin^1/2^) is the intraparticle diffusion rate constant. The slope of the linear plot of *q*_*t*_ against *t*^1/2^ gives the value of *K*_idm_.

## 3. Results and Discussions

### 3.1. Characterization

#### 3.1.1. Determination of Water and Amine Content in FGCX

The water content present in GFCX was found to be 74.6%. This water content is vital for the effective transport of metal ions to adsorption site. Consequently, in determining the amine concentration of FGCX the pH as a function of the amount of acid added was studied and the point of inflection from the graph directed to the *x*-axis gave the value of amine concentration. The amine concentration of FGCX was found to be 5.3 mmol/g.

### 3.2. XRD Result

**Figure 3:**
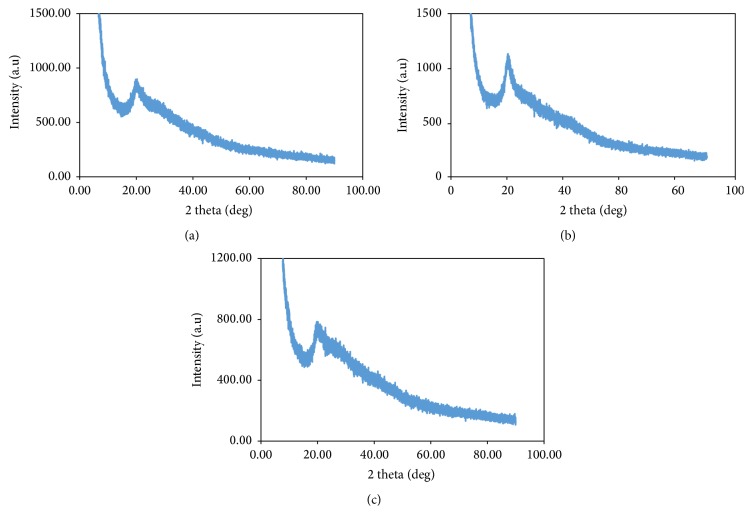
XRD of CX, CCX, and FGCX, respectively.

The crystallinity of the three sets of beads was studied with X-ray diffraction analyser. Figures 3(a), 3(b), and 3(c) show the diffraction pattern of chitosan, cross-linked chitosan, and grafted cross-linked chitosan beads, respectively. The non-cross-linked chitosan is said to be crystalline in nature; this crystalline nature prevents the amine group from binding efficiently with adsorbate [9]. A common feature of 2*θ* = 20° which corresponds to 110 planes of chitosan was observed in Figures 3(a) and 3(b); this is because it is possible to modify chitosan and still retain some of its properties. However, in Figure 1(c) there was a slight shift in the peak to 2*θ* = 25° due to copolymer formation which provides evidence of successful grafting. A similar trend was reported by Igberase et al. [5]. Also in Figure 3(c), the intensity reduced drastically because some of the crystalline chains have been eliminated during grafting process.

### 3.3. SEM Result

**Figure 4:**
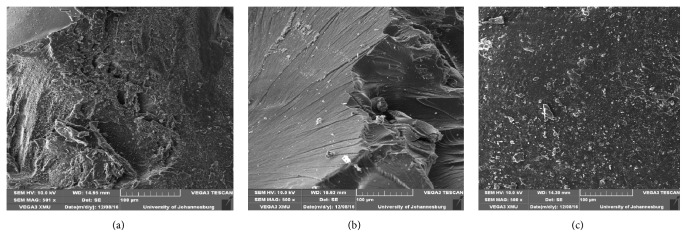
SEM analysis of chitosan, cross-linked chitosan, and grafted cross-linked chitosan beads, respectively.

SEM was utilized to analyse the morphology and changes of chitosan after cross-linking and grafting. SEM images of the different set of beads are presented in Figures 4(a), 4(b), and 4(c). The surface of CCX appears to be more visible and smooth as compared to CX due to the reaction between native chitosan beads and glutaraldehyde and as such glutaraldehyde has been chemically bonded with chitosan. The grafting of 4-aminobenzoic acid onto the backbone of cross-linked beads lead to the evenness of the surface.

### 3.4. FTIR Result

**Figure 5:**
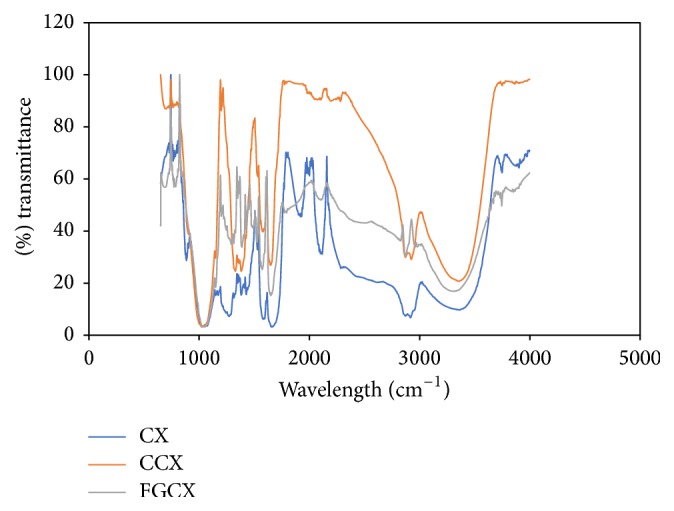
FTIR of chitosan and cross-linked chitosan beads.

The infrared spectroscopy was used to provide evidence of comparable difference between the three sets of beads which are CX, CCX, and FGCX (Figure 5). The broad band of chitosan, cross-linked chitosan, and grafted cross-linked chitosan at wavelengths 3384 cm^−1^, 3385 cm^−1^, and 3389 cm^−1^ indicates the presence of exchangeable protons which is from alcohol and amine group. The slight shift in band may be due to exchangeable protons in the modification process. The C–H stretch at wavelengths of 2918 cm^−1^, 2926 cm^−1^, and 2875 cm^−1^ corresponds to chitosan, cross-linked chitosan, and grafted cross-linked chitosan, respectively [30]. All three sets of beads had a common wavelength of 1000 cm^−1^ which indicates C–O stretching vibration, since it is possible for an adsorbent to retain some of its properties after modification [5]. The sharp peaks at wavelength 1217 cm^−1^ and 1508 cm^−1^ in the cross-linked chitosan beads correspond to C–N stretching vibration and N=O stretching vibration, respectively. The IR spectra of the cross-linked chitosan beads showed increase in intensity between wavelength 1217 cm^−1^ and 1653 cm^−1^ as compared to chitosan beads. Subsequently the grafted cross-linked chitosan showed increase in intensity between wavelength 1195 cm^−1^ and 1653 cm^−1^. This provides evidence of successful modification.

### 3.5. TGA Result

**Figure 6:**
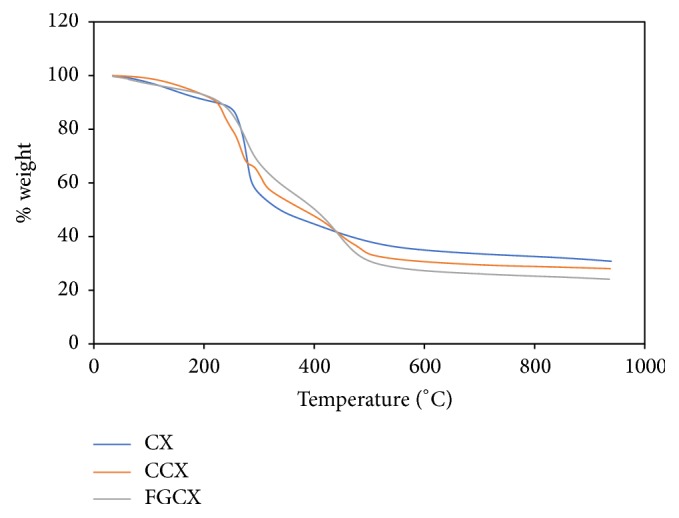
TGA of CX, CCX, and FGCX.

TGA was used to study the thermal properties when heat is applied in the three set of beads. A plot of weight % versus temperature was done to study the thermal stability of the set of beads.  Figure 6 depicts the stages involved in the thermal degradation of the sets of beads. The decomposition of CX took place in 3 stages; in the first stage there was 6% weight loss between temperatures of 34 and 148°C which corresponds to the removal of water from the adsorbent [31]. The second stage started at 250°C up to 320°C with 36% weight loss. This loss of weight is ascribed to the dehydration of the saccharide rings, depolymerisation, and decomposition of the acetylated and deacetylated units of adsorbent [32]. In the third stage there was 51% loss of weight above 400°C. This stage is the decomposition of non-cross-linked chitosan.

The CCX showed a 5% weight loss at temperatures between 34 and 141°C during the first stage. The second stage started at 227°C and continued up to 330°C with 31% weight loss. In the third stage, there was 10% weight loss above 500°C. This loss of weight in the 1st, 2nd, and 3rd stages corresponds to removal of surface water, depolymerisation, and decomposition of the acetylated and deacetylated units of adsorbent and decomposition of cross-linked chitosan, respectively [31, 32].

The FGCX showed a 10% weight loss at temperatures between 51 and 150°C in the first stage of degradation. The second stage started at 280°C and continued up to 398°C with 24% weight loss. In the third stage, there was 15% weight loss above 500°C. This loss of weight in the 1st, 2nd, and 3rd stages corresponds to removal of surface water, depolymerisation, and decomposition of the acetylated and deacetylated units of adsorbent and decomposition of grafted cross-linked chitosan, respectively [31, 32].

### 3.6. Effect of Solution pH

**Figure 7:**
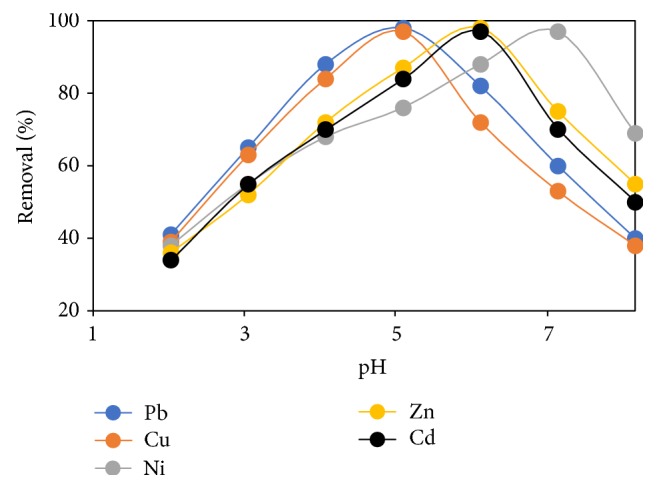
Effect of pH on percentage removal of heavy metal ions by FGCX (conditions: 7 g/l FGCX; contact time: 70 min; Temperature: 25°C; initial concentration: 0.8 mmol/L; agitation speed: 120 rpm).

Metal binding efficiency of amino-functionalized ligand is greatly affected by pH, since it influences the surface charge of ligands and ionisation degree [33]. It also impacts on the level of adsorbate precipitation and the nature of structures formed between adsorbate and adsorbents. A plot of percentage removal against solution pH was made to obtain the optimum pH for the binding of heavy metal ions onto grafted cross-linked chitosan beads, as presented in Figure 7. In this figure there was a noticeable reduction in percentage removal at lower pH values which is mainly due to the protonation of the amine group that creates an electrostatic repulsion between the metal ions in solution and the amine group of chitosan. As the pH is increased the surface of the beads becomes negatively charged due to deprotonation reaction [9]. Consequently, the repulsive force that exists between metal ions in solution and the amine group of chitosan decreases, thus increasing the removal of metal ions in solution until an optimum value is achieved. At pH above the optimum value insoluble metal hydroxide begins to precipitate from the solution resulting in the decrease of metal ions removal from the solution.

### 3.7. Effect of Contact Time

**Figure 8:**
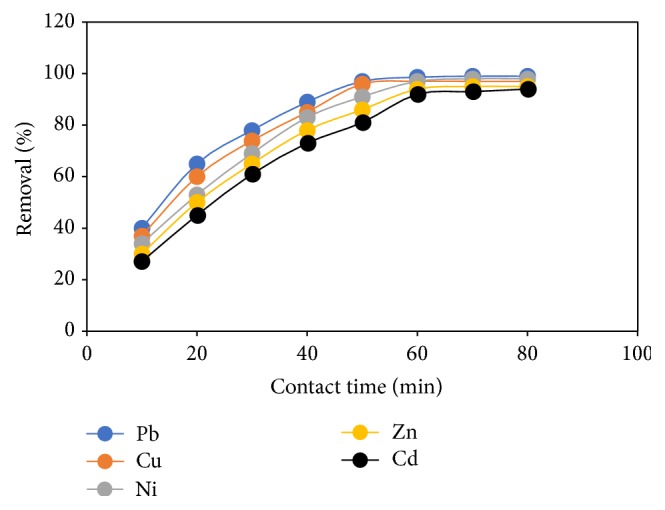
Effect of contact time on percentage removal of heavy metal ions by FGCX (conditions: 7 g/l FGCX; pH: Pb = 5, Cu = 5, Ni = 7, Zn = 6, and Cd = 6; Temperature: 25°C; initial concentration: 0.8 mmol/L; agitation speed: 120 rpm).

In any given adsorption system it is vital to establish the time dependence of the system in achieving equilibrium.  Figure 8 depicts the effect of contact time on the adsorption of heavy metals from a single component solution and time range of 10–80 mins. It was observed that 45 min was sufficient to establish equilibrium for Pb, Cu, and Ni while it took 55 min for Zn and Cd. The adsorption was very fast at the initial stage for all metal ions investigated due to sufficient and well aligned site available for the binding of the considered metal ions but became slower until equilibrium is reached since the binding sites have been covered with metal ions which causes repulsion with increase in time [9, 34]. This result is important because longer contact time between the beads and metal ions in solution can consume energy and hence increase cost of treatment.

### 3.8. Effect of Temperature

**Figure 9:**
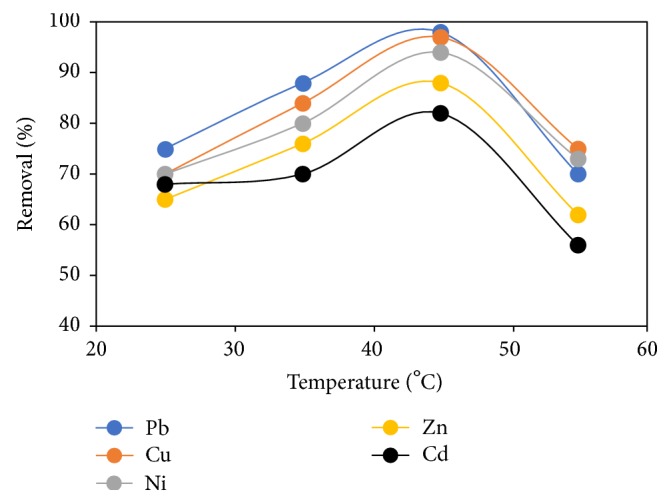
Effect of contact time on percentage removal of heavy metal ions by FGCX (conditions: 7 g/l FGCX; pH: Pb = 5, Cu = 5, Ni = 7, Zn = 6, and Cd = 6; contact time: 60 min; initial concentration: 0.8 mmol/L; agitation speed: 120 rpm).

Temperature as an important parameter in adsorption process is directly linked to the kinetic energy of adsorbent in the solution [35]. The effect of temperature was studied at a temperature range of 25–65°C and a plot of percentage removal against temperature (Figure 9) was made to elucidate the observation. It was shown that a rise in temperature expedites the binding of metal ions until an optimum of 45°C is reached. This finding is due to the fact that as the temperature rises the kinetic energy also rises which then promotes the accessibility of metal ions onto adsorbents and, in the process, reduces the time to reach adsorption equilibrium [36, 37]. Also, the reaction of amino groups with ions is exothermic and the process for the distribution of metal ions into the FGCX pores is endothermic and this positive enthalpy change is greater than the negative enthalpy change due to the formation of complexes between the amino group of FGCX and considered metal ions in solution leading to an overall positive enthalpy change [25]. Hence a rise in temperature is vital for the adsorption of metal ions onto FGCX. Consequently, as the temperature exceeds that of the optimum, deterioration of the adsorbent may occur leading to reduced removal of metal ions. This result is favourable by virtue of cost effectiveness.

### 3.9. Effect of Initial Concentration

**Figure 10:**
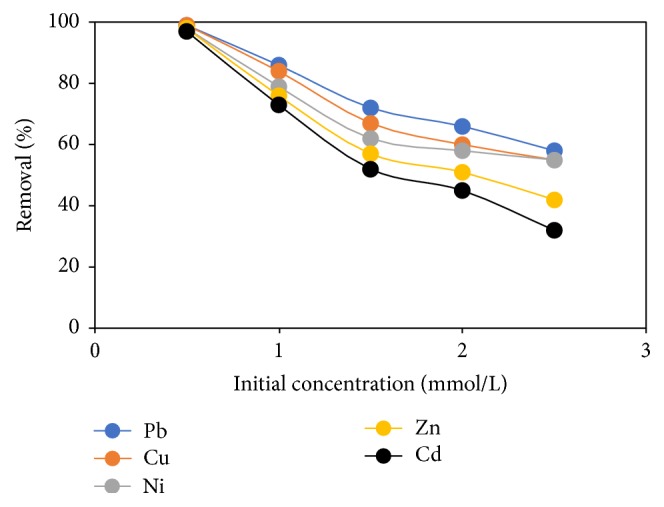
Effect of initial concentration on percentage removal of heavy metal ions by FGCX (conditions: 6 g/l FGCX; pH: Pb = 5, Cu = 5, Ni = 7, Zn = 6, and Cd = 6; contact time: 60 min; agitation speed: 120 rpm; temperature: 45°C).

The effect of initial metal concentration is of significance in adsorption studies, since it depends on several parameters such as the type of metal and the liquid medium, the presence of competing cations, the availability of the functional groups in the adsorbent surface, and the ability of these groups to attach metal ions [38–40].  Figure 10 explores the percentage removal of heavy metal ions as a function of initial concentration. It was observed that at lower initial concentration of 0.5 mmol/L the %* R* of Pb, Cu, Ni, Zn, and Cd were 99.9, 99.5, 98.6, 98, and 97.8%, respectively. At higher initial concentration of 2.5 mmol/L the %* R* were Pb (58%), Cu (55%), Ni (55%), Zn (42%), and Cd (32%). This may be due to the fact that at lower initial concentration the ratio of metal cations to FGCX mass is low; a rise in initial concentration implies that additional metal ions are present in the mixture and hence more ions are attached to same quantity of FGCX which results in saturation of the FGCX adsorbent causing a decrease in %* R*. Also, at higher initial concentration the driving force to overcome the mass transfer opposition for the movement of the metal ions from the mixture to the adsorbent surface increases and, in this case, higher concentration would lead to saturation of the adsorbent surface [41].

### 3.10. Effect of Adsorbent Dose

**Figure 11:**
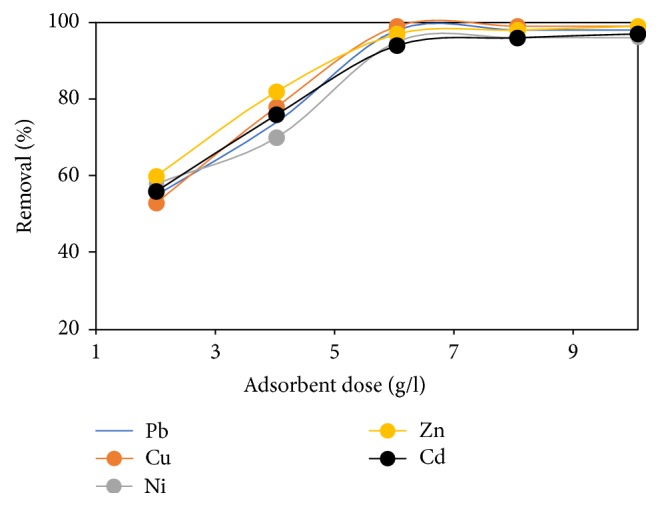
Effect of FGCX dose on percentage removal of heavy metal ions (conditions: pH: Pb = 5, Cu = 5, Ni = 7, Zn = 6, and Cd = 6; contact time: 60 min; initial concentration: 0.5 mmol/L; agitation speed: 120 rpm; temperature: 45°C).

The adsorbent type and functional group present in the adsorbent has intense effect on adsorption, since it determines the availability of binding sites.  Figure 11 illustrates the effect of FGCX dose on the %* R* of metal ions. There was a sharp increase in %* R* of metal ions at FGCX dose range of 2 to 6 g/l and increasing the FGCX dose above 6 g/l did not cause any further changes in the %* R* of metal ions. The observed behaviour occurred because at the initial stage there were sufficient binding sites for the complexation of metal ions and increasing the dose beyond 6 g/l resulted in the establishment of equilibrium between the metal ions bounded to FGCX and those remaining unabsorbed in the mixture [9, 42].

### 3.11. Effect of Agitation Speed

**Figure 12:**
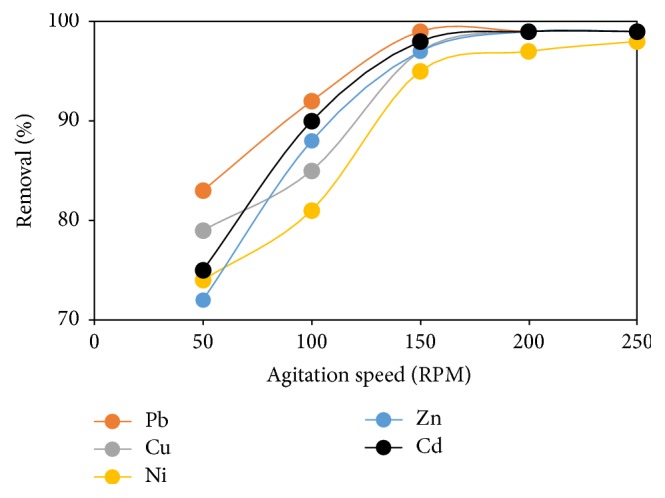
Effect of agitation speed on percentage removal of heavy metal ions by FGCX (conditions: 6 g/l FGCX; pH: Pb = 5, Cu = 5, Ni = 7, Zn = 6, and Cd = 6; contact time: 60 min; initial concentration 0.5 mmol/L; temperature: 45°C).

Agitation speed is an important parameter that is mostly neglected by some researchers in adsorption studies. Park et al., [43], reported that the application of appropriate agitation speed increases the movement of metal ions in the mixture, reducing the mass transfer resistance in the process.  Figure 12 depicts the impact of agitation speed on %* R* of metal ions by FGCX at agitation speed range of 50–250 rpm. It was observed that agitation speed has a positive impact on metal ions removal by FGCX. Consequently, there was a fast increase in metal ions removal up to 150 rpm and thereafter it became constant. This is because at high agitation speed the boundary layer becomes thinner which eventually influence the speed of distribution of metal ions through the boundary layers [44]. However, a further increase beyond 150 rpm would lead to saturation of the adsorption site.

### 3.12. Effect of Ionic Strength

**Figure 13:**
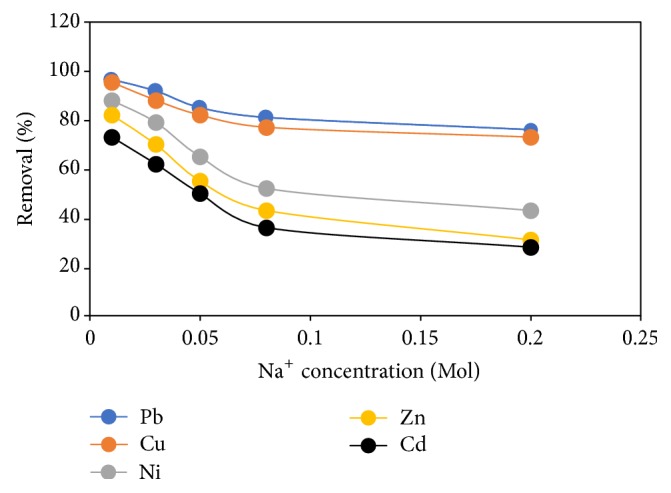
Effect of ionic strength on percentage removal of heavy metal ions by FGCX (conditions: 6 g/l FGCX; pH: Pb = 5, Cu = 5, Ni = 7, Zn = 6, and Cd = 6; contact time: 60 min; initial concentration 0.5 mmol/L; temperature: 45°C; agitation speed: 150 RPM).

Wastewater usually comprises different cations and anions that can hinder the efficient binding of metal ions in solution.  Figure 13 elucidates the effect of ionic strength on the adsorption of metal ions onto FGCX. In this plot there was a decrease in %* R* with increase in the concentration of NaNO_3._ This observation shows that the interaction between the amine group of FGCX and the metal cations is ionic in nature and raising the ionic strength reduces their movement to the solid surface [4]. Among the five metals investigated Pb and Cu proved to have higher binding strength even when the concentration of Na^+^ was increased. This may be because some binding sites have the potential to attach specific metal ions [45].

### 3.13. Isotherm Result

**Figure 14:**
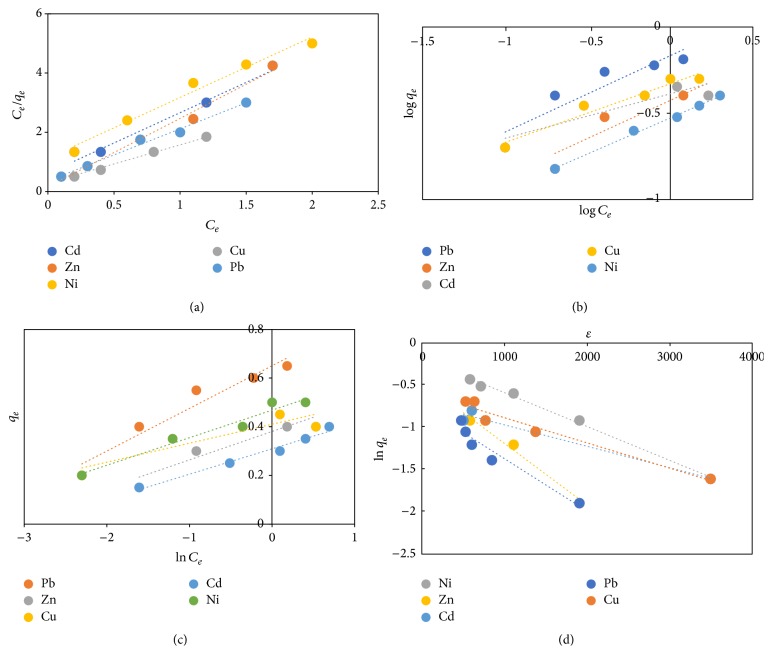
Langmuir, Freundlich, Temkin, and DKR isotherm models, respectively, at temperature of 45°C.

The closer *R*^2^ values are to one the best the model fits [9]. The parameters of the models are presented in Table 1, while Figures 14(a), 14(b), 14(c), and 14(d) depict the plot for Langmuir, Freundlich, Temkin, and DKR models, respectively.

The Langmuir model was efficient in analysing adsorption data of all the metal ions studied with *R*^2^ values ≥ 0.98. *Q*_*m*_ values for the binding of metal ions onto FGCX followed a decreasing sequence of Pb (3.96) > Cu (2.89) > Ni (2.8) > Cd (1.82) > Zn (1.76 mmol/g), whereas *K*_*L*_ which determines the affinity of binding proved that the GFCX sites had greater affinity for Cu and the trend followed a decreasing order of Cu (3.71) > Pb (2) > Ni (1.33) > Cd (1.08) > Zn (0.86 l/mmol). This trend can be explained in terms of electronegativity of metal ions, since adsorption of these metal ions is also due to ion exchange at the surface. Therefore, more electronegative metal ions will exhibit a higher tendency of adsorption. The electronegativities of Pb, Cu, Ni, Cd, and Zn per Pauling scale are 1.87, 1.9, 1.8, 1.7, and 1.6, respectively, which agrees with the order of affinity. Based on this study it can be said that the affinity of metal ions onto FGCX is also believed to be a function of electronegativity. The *R*_*L*_ values for Cu, Pb, Ni, Cd, and Zn were 0.35, 0.5, 0.6, 0.65, and 0.7, respectively, and these values signify favourable adsorption process.

The Freundlich model was successful in analysing experimental data of Ni and Zn ions onto FGCX while that of Pb, Cu, and Cd did not fit the model very well. The *K*_*f*_ values decreased in the order of Ni (2.13) > Zn (2.05) > Pb (1.79) > Cu (1.7) > Cd (1.46 mmol/g); the *n* values indicated favourable adsorption for the considered metal ions.

The data for the adsorption of Pb, Ni and Zn onto FGCX was in agreement with Temkin model with *R*^2^ value ≥ 0.98 in contrast to the *R*^2^ value (≥0.85) obtained for the adsorption of Cu and Cd.

The DKR model was successful in describing experimental data for the considered metal ions with *R*^2^ value ≥ 0.98. The adsorption capacity (*X*_*m*_) was found to be 3.92, 2.75, 2.85, 1.79, and 1.77 mmol/g for Pb, Cu, Ni, Zn, and Cd, respectively. The adsorption energy values for these ions are, respectively, 14.55, 13.42, 13.11, 10.21, and 9.82 kJ/mol. These values indicate the process can also be described by ion exchange mechanism.

### 3.14. Thermodynamic Parameters

**Figure 15:**
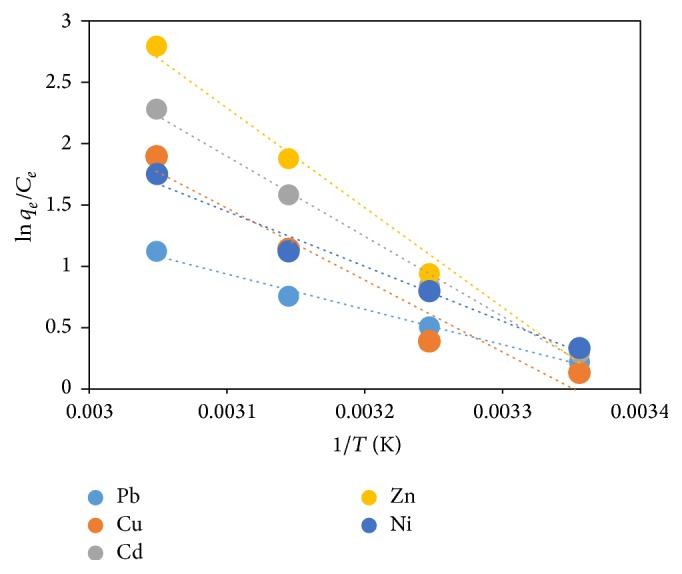
Thermodynamic plot of ln⁡*K* versus 1/*T* for metal ions binding onto FGCX.

The plot of ln⁡*K* versus 1/*T* for the adsorption of Pb, Cu, Ni, Zn, and Cd onto FGCX is presented in Figure 15.  Table 3 provides the parameters for thermodynamic studies. The free energy change (Δ*G*^*o*^) obtained during the absorption reaction at temperatures of 25, 35, 45, and 55°C was all negative and this indicates that the adsorption of heavy metals onto FGCX is spontaneous and favourable. A similar finding was reported by Zawani et al. [46]. Also, increase in negative values of Δ*G*^*o*^ as temperature increases indicates greater driving force for binding of metal ions. The positive value of Δ*H*^*o*^ indicates that the adsorption process is endothermic in nature. This finding is in agreement with the result presented by Liu et al. [17]. The positive value of Δ*S*^*o*^ indicates the increased randomness at the solid-solution interface during the adsorption of heavy metal ions onto FGCX [17].

### 3.15. Kinetic Parameters

**Figure 16:**
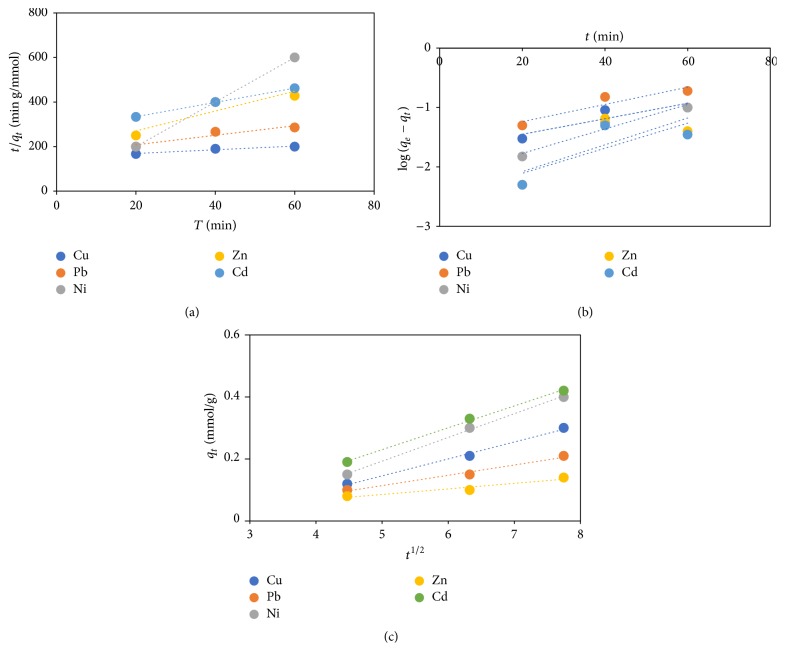
Pseudo-first-, pseudo-second-, and intraparticle diffusion plot, respectively.

The straight-line plot of the pseudo-first- and pseudo-second-order and intraparticle diffusion model for the binding of assessed metal ions (Pb, Cu, Cd, Zn, and Ni) onto FGCX is presented in Figures 16(a), 16(b), and 16(c), and the values of the parameters in each case are presented in Table 4. It is evident that the *R*^2^ values for the pseudo-second-order and intraparticle model are higher and closer to one in comparison with pseudo-first-order model. This observation indicates that the pseudo-second-order and intraparticle model best describe the kinetic data of the assessed metal ions binding onto FGCX. Also, the pseudo-second rate constant (*K*_2_) followed a decreasing sequence of Pb (7.34) > Cu (5.82) > Ni (5.12) > Zn (3.21) > Cd (2.56) in g/mmol·min. This suggests that the chemical interaction is dependent on the affinity of metal ions to interact with the amino group of FGCX. A similar report was presented by Bulgariu et al., [26].

### 3.16. Reusability of the Spent FGCX

Adsorbent reuse is an important aspect of adsorption studies in that its reusability helps lower the processing cost. The recovery of the considered metal ions and subsequent usage of the adsorbent were made possible with 0.5 M HCl and contact time of 180 min; the recovery efficiencies of the metal ions from its spent FGCX were 98.8, 97.5, 97.9, 98.3, and 97.7 for Pb, Cu, Ni, Zn, and Cd, respectively. There was no loss in the mass of the regenerated beads.

### 3.17. Reaction Mechanism of Chitosan

Most researchers support that the amine group of chitosan is the main reactive site for metal ions, though hydroxyl groups may contribute to binding of metal ions [9]. However, of the free amino groups only some are accessible to metal binding, since some of these amine sites interact with hydrogen ions at lower pH. These reactive groups may react with metal ions through different mechanisms such as chelation and electrostatic depending on factors such as the type of metal, the pH, fraction of deacetylated units (free amine groups), polymer chain length, crystallinity, molecular weight, conditioning of polymers, physical form of chitosan, solution pH, type and concentration of the acid used for solution, composition of solution, metal ion selectivity and speciation, and the matrix of the solution [47]. The amine group initiates a coordinate bond with the metal ions; the bond is formed between the free electron pairs of the nitrogen in the amine group and the void orbitals of the metal. Also, the free electron doublet of nitrogen on amine groups is responsible for the adsorption of metal cations at pH near neutrality and at lower pH value, where protonation of the amine group takes place; the polymer attains cationic groups which can bind anions through electrostatic interactions [48].

## 4. Conclusion

A promising adsorbent was developed by grafting 4-aminobenzoic acid onto the backbone of cross-linked chitosan beads which reduced its crystallinity and expanded the polymer network for easy accessibility of the considered metal ions to binding site. Comprehensive investigation was performed in order to access the effect adsorption parameters have on the binding of metal ions onto FGCX. The adsorption of Pb, Cu, Ni, Zn, and Cd was dependent on pH, contact time, adsorbent dosage, initial concentration, agitation speed, and ionic strength. The binding of these metal ions was observed to be a feasible, spontaneous, and endothermic process and corresponds with the Langmuir and DKR isotherm model, while some metals were in agreement with Freundlich and Temkin model. The binding was also observed to be in agreement with pseudo-second-order and intraparticle diffusion model. The result indicated that binding of these metal ions is mainly controlled by chemisorption, electrostatic attraction, or ion exchange. At industrial level, this developed adsorbent with high amino content can be an excellent candidate for the adsorption of metal ions even the ions not mentioned in this study.

## Figures and Tables

**Table 1:** Langmuir, Freundlich, Temkin, and DKR isotherm parameters for binding of considered metal ions onto FGCX at temperature of 45°C.

Ions	Langmuir parameters			Freundlich parameters			Temkin parameters			DKR parameters		
	*Q*_*m*_	*K*_*L*_	*R* ^2^	*n*	*K*_*f*_	*R* ^2^	*A*	*B*	*R* ^2^	*X*_*m*_	*E*	*R* ^2^
	(mmol/g)	(l/mmol)			(mmol/g)		(l/mmol)	(J/mol)		(mmol/g)	(kJ/mol)	
Pb	3.96	2	0.99	2.70	1.79	0.86	2.99	9.11	0.98	3.92	14.55	0.99
Cu	2.88	3.71	0.99	2.22	1.70	0.86	3.42	10.33	0.85	2.75	13.42	0.99
Ni	2.80	1.33	0.98	3.42	2.13	0.98	1.52	11.21	0.98	2.85	13.11	0.97
Cd	1.82	1.08	0.99	1.98	1.46	0.87	1.20	10.56	0.88	1.79	10.21	0.99
Zn	1.76	0.86	0.99	2.5	2.05	0.99	0.92	9.98	0.99	1.77	9.82	0.99

**Table 2:** Comparison of adsorption capacity of FGCX onto Pb, Cu, Ni, Cd, and Zn with other absorbent.

Heavy metal ions	Adsorbent	*Q*_*m*_ (mmol/g)	Temperature	Reference
Lead	Mesoporous silicas	2.86	25	[25]
	Romania peat	0.20	20	[26]
	Lignin	0.43	20	[27]
	Cross-linked CMCS resin	1.89	25	[28]
	FGCX	3.96	45	This study

Copper	Mesoporous silicas	2.34	25	[25]
	Cross-linked CMCS resin	1.82	25	[28]
	Lignin	0.36	20	[27]
	FGCX	2.88	45	This study

Nickel	Lignin	0.10	20	[27]
	Cross-linked CMCS resin	0.78	25	[28]
	Alginate microcapsules	0.52		[29]
	FGCX	2.80	45	This study

Cadmium	Mesoporous silicas	1.71	25	[25]
	Romania peat	0.39	20	[26]
	Lignin	0.23	20	[27]
	FGCX	1.82	45	This study

Zinc	Mesoporous silicas	1.36	25	[25]
	Lignin	0.17	20	[27]
	Cross-linked CMCS resin	0.74	25	[28]
	FGCX	1.76	45	This study

**Table 3:** Thermodynamic parameters for the adsorption of heavy metal ions onto FGCX at an initial concentration of 0.5 mmol/l.

Metal ions	Δ*S*^*o*^	Δ*H*^*o*^	∆*G*^*o*^ (kJ/mol)				*R* ^2^
	(kJ/mol/k)	(kJ/mol)	*T* = 298 K	*T* = 303 K	*T* = 318 K	*T* = 328 K	
Pb	57.23	0.23	−1.52	−1.34	−4.45	−4.98	0.99
Cu	64.78	0.26	−1.25	−1.65	−3.97	−4.91	0.99
Ni	67.12	0.20	−2.31	−6.92	−8.78	−9.56	0.98
Zn	54.26	0.32	−1.20	−2.32	−4.75	−6.43	0.99
Cd	68.45	0.27	−2.58	−4.41	−8.54	−9.43	0.97

**Table 4:** Kinetic parameters for the binding of considered metal ions onto FGCX.

Metal ions	Pseudo-first-order parameters			Pseudo-second-order parameters			Intraparticle diffusion parameters	
	*K*_1_	*q*_*e*_	*R* ^2^	*K*_2_	*q*_*e*_	*R* ^2^	*K*_idm_	*R* ^2^
	(min^−1^)	(mmol/g)		(g/mmol·min)	(mmol/g)		(mmol/gmin^1/2^)	
Pb	0.03	1.59	0.88	7.34	3.54	0.98	3.43	0.97
Cu	0.01	1.16	0.81	5.28	3.87	0.99	4.18	0.99
Ni	0.02	1.90	0.83	5.12	3.12	1.00	2.97	0.99
Zn	0.03	2.2	0.83	3.21	3.99	0.99	2.56	0.98
Cd	0.05	1.85	0.73	2.56	3.52	0.99	3.78	0.99
